# Performance Enhancement for B5G/6G Networks Based on Space Time Coding Schemes Assisted by Intelligent Reflecting Surfaces with Higher Modulation Orders

**DOI:** 10.3390/s24196169

**Published:** 2024-09-24

**Authors:** Mariam El-Hussien, Bassant Abdelhamid, Hesham Elbadawy, Hadia El-Hennawy, Mehaseb Ahmed

**Affiliations:** 1 Electronics and Communications Department, Faculty of Engineering Science and Arts, Misr International University, Cairo 11828, Egypt; mehasb.ahmed@miuegypt.edu.eg; 2 Electronics and Communications Department, Faculty of Engineering, Ain Shams University, Cairo 11517, Egypt; bassant.abdelhamid@eng.asu.edu.eg (B.A.); hadia.elhennawy@gmail.com (H.E.-H.); 3 Network Planning Department, National Telecommunications Institute, Cairo 11768, Egypt; heshamelbadawy@ieee.org

**Keywords:** Intelligent Reflecting Surfaces (IRS), Multiple-Input Single-Output (MISO), Orthogonal Space-Time Block Code (OSTBC), Bit Error Rate (BER)

## Abstract

Intelligent Reflecting Surfaces (IRS) and Multiple-Input Single-Output (MISO) technologies are essential in the fifth generation (5G) networks and beyond. IRS optimizes the signal propagation and the coverage and is a viable approach to address the issues caused by fading channels that limits the spectral efficiency, while MIMO enhances data rates, reliability, and spectral efficiency by using multiple antennas at both transmitter and receiver ends. This paper proposes an IRS-assisted MISO system using the Orthogonal Space-Time Block Code (OSTBC) scheme to enhance the channel reliability and reduce the Bit Error Rate (BER) in wireless communication systems. The proposed system exploits the benefits from the transmit diversity gain of the OSTBC scheme as well as from the bit energy to noise power spectral density (E_b_/N_o_) improvement of the IRS technology. The presented work explores these combined technologies across different modulation schemes. The obtained results outperform the similar previously published works by considering higher-order modulation schemes as well as the deployment of rate ¾ OSTBC-assisted IRS. Moreover, the obtained results demonstrate that the integration of OSTBC with IRS can yield significant performance improvements in terms of E_b_/N_o_ by 7 dB and 13 dB when using 16 reflecting elements and 64 reflecting elements, respectively.

## 1. Introduction

Significant throughput and reliability are considered basic requirements in the current era of communication. In the fifth generation (5G) and beyond 5G era, massive data storage, faster data processing, and 100% uptime availability are essential, for which mobile communication is the basic source of data transfer due to more mobility and less infrastructure. The success of any communication system depends on the channel through which it was transmitted. Due to multipath fading, degradation of the channel quality affects the system throughput and the reliability. Thus, it is necessary to improve the system reliability and throughput to fulfill the demand of the new era.

Massive Multiple-Input Multiple-Output (mMIMO) systems are a wireless communication technology that use multiple antennas on both the transmitting and receiving sides. Through the utilization of this methodology, MIMO systems offer numerous benefits, such as enhanced capacity, enhanced reliability, and increased spectral efficiency [1,2].

In the field of MIMO communications, three primary concepts can be identified: spatial multiplexing, transmit diversity, and spatial modulation [3]. Spatial multiplexing implies the technique of concurrently transmitting numerous symbols from multiple pairs of antennas over a single-channel use [4]. Spatial multiplexing is utilized as an approach for enhancing both the data rate and the spectral efficiency of the system. On the other hand, transmit diversity refers to the simultaneous transmission of delayed and different versions of the symbols by various antennas, which is then equalized at the user equipment (UE) to provide diversity gain [5]. Lastly, Spatial Modulation (SM) is a cost-effective technology that activates a single Radio Frequency (RF) chain at the transmitter. SM utilizes the transmit antenna indexes as an additional information carrier [6]. The SM was introduced by Mesleh et al. in [7,8]. The improvement of data rate provided by MIMO systems comes at the expense of increased complexity, size, and higher hardware costs. In this paper, MIMO is used as transmit diversity and one antenna is used at the UE, so the MISO system is considered.

In wireless communication systems, the utilization of Space-Time Block Code (STBC) is employed as an approach to improve the reliability and performance of data transmission over multiple antennas. The STBC protocol involves the distribution of data symbols across multiple transmit antennas, which are then transmitted over a specified period of time. This allows the receiver to take advantage of spatial diversity and effectively mitigates fading and multipath effects [9]. The Alamouti scheme was the first orthogonal STBC (OSTBC) to provide full transmit diversity for systems with two transmit antennas [10]. By utilizing orthogonal coding schemes like Alamouti coding, STBC ensures that the transmitted signals are decoupled at the receiver, enabling reliable detection and decoding.

The receivers have the ability to detect multiple versions of the originally transmitted signal. This repetition of transmitted signals can significantly enhance the performance of the communication system. It leads to improved reception quality at the receivers. Additionally, the transmitting antennas will have a reduction on the overall power-transmission levels, leading to power enhancement at the transmitters. Furthermore, through cooperative communication operations, the radio system can effectively minimize the Bit Error Rate (BER). This, in turn, enhances the overall reliability of the system. Ensuring system reliability is of paramount importance in radio systems. STBC are orthogonal as originally introduced by Alamouti. OSTBC is employed for simple, linear, and optimal decoding at the receiver. However, the existence of quasi-orthogonal STBC (QO-STBC) introduces inter-symbol interference (ISI).

Intelligent Reflecting Surfaces (IRS) have emerged as a promising technology for enhancing wireless communication systems [11]. IRS is an array of smart reflecting surfaces that are separated by a distance equal to half of the wavelength [12]. IRS technology was first proposed in [13] as a promising technology for the future sixth generation (6G) wireless communications and beyond systems. One crucial aspect to highlight is that the IRS enables the gNodeB to support the UE by establishing a virtual line-of-sight (LOS) path when the physical LOS path is obstructed [14]. This capability of the IRS is of utmost significance. The deployment of this technology is versatile, as it can be implemented on various structures such as buildings, walls, ceilings, and even underground tunnels [15].

IRS elements may be active or passive. Passive IRS has the ability to independently modify the incident signal in various ways. These modifications can involve changes in phase, amplitude, frequency, or even polarization [16]. IRS enables efficient signal reflection, beamforming, extending signal coverage, and interference mitigation [17]. However, unlike passive IRS, active IRS can also amplify the reflected signal. This is achieved through the integration of active reflection amplifiers within each active IRS element [18]. Sophisticated beamforming and precoding were studied in [19].

IRS is versatile and compatible with various communication technologies, such as millimeter-wave and terahertz communications, Simultaneous Wireless Information, and Power Transfer (SWIPT), Unmanned Aerial Vehicle Networks (UAV), MIMO systems, cell edge communication, physical layer security, and Non-Orthogonal Multiple Access (NOMA) [20].

The implementation of the Smart electromagnetic environment, by introducing several active and passive devices that are able to reach blind spots or to cover desired areas without increasing the number of base stations, is very challenging [21]. Among them, Smart Electromagnetic Skins (SESs), are very thin passive surfaces capable of enabling non-specular reflection to reach blind spots or cover target areas [22].

In contrast to traditional Access Points (APs) or relays, IRS operates solely through passive reflection and does not necessitate high energy consumption or costly hardware [23]. This characteristic enables IRS to be deployed in a dense manner, offering substantial enhancements to the network performance [24]. In the next-generation wireless communication system, the utilization of IRS technology plays a crucial role in enhancing the performance of the physical layer.

In [25], the authors proposed an IRS-assisted millimeter-wave massive MIMO system with transmit antenna selection using the Alamouti scheme and hybrid analog-digital beamforming to achieve better beamforming gain. Moreover, the use of IRS also leads to significant BER improvements. The Alamouti technique is used in the transmitting side where only two antennas are used. The authors in [26] proposed the usage of IRS to improve the BER of the system to eliminate the effect of electromagnetic interference. In addition, they used STBC using three transmitting antennas to compensate the bad performance of the system. In [27], the authors introduced two MIMO systems assisted by IRS to improve the spectral efficiency by redesigning the classical Alamouti scheme and vertical Bell Labs Layered Space-Time (V-BLAST) for the IRS-aided communication scenario. Hence, the proposed methodology of [27] demonstrated that these redesigned systems achieved significantly enhanced end-to-end performance, highlighting the potential of IRS in boosting the spectral efficiency. In [28], the authors introduced the design of the IRS-aided QO-STBC scheme, where Quasi-Orthogonal STBC (QOSTBC) is applied on IRS elements instead of transmitting antennas. The proposed approach in [29] utilizes a phase-programmable meta-surface, which is fabricated and evaluated through a prototype system. The prototype system successfully achieves real-time RIS-based Alamouti space-time transmission over the air, demonstrating the effectiveness of the proposed approach. The authors in [30] proposed the Alamouti STBC transmitter diversity technique along with the IRS-assist MISO wireless communication system to mitigate the effect of phase shift noise and quantization noise. The authors in [31] proposed an IRS-based Alamouti scheme that allows the IRS to transmit coded information while reflecting the incident Space Shift Keying (SSK) signals. In [32], the authors proposed the IRS-assisted received spatial modulation (RSM) scheme with Alamouti STBC. In [33], they proposed an Alamouti STBC, generalized spatial modulation, and IRS incorporated into a SWIPT system to increase the spectral efficiency.

These papers did not consider IRS-aided OSTBC for four antennas with code rate ¾. Different from the previous-mentioned works, this paper investigates the deployment of higher-order modulation schemes for an Alamouti and OSTBC IRS-aided system compared to the conventional non-IRS system for four transmit antennas. The main contribution of the presented work can be summarized in the following points:

- The paper employs a combination of OSTBC and IRS technology, to reduce the required bit energy to noise power spectral density (E_b_/N_o_) in case of the MISO system.
- In contrast to prior work, OSTBC is utilized instead of QOSTBC to prevent ISI.
- Unlike the previous work, the paper utilizes OSTBC with a ¾ code rate combined with IRS.
- A detailed mathematical model is introduced for OSTBC combined with IRS.
- The proposed systems of OSTBC are simulated in case of different modulation techniques (QPSK, 16 QAM, 64 QAM, and 256 QAM).
- The system performance is evaluated and compared in both cases with IRS and without IRS to assess the benefits of the IRS integration.

The obtained results show that by employing OSTBC as well as increasing the number of transmitting antennas, the system’s performance improves. In addition, the BER decreases by increasing the number of reflecting elements of the IRS due to the constructive reflections from the IRS elements.

The rest of the paper is organized as follows: In Section 2, the system model of OSTBC-assisted IRS is presented. The mathematical model for Alamouti and OSTBC-assisted IRS is presented in Section 3. Section 4 presents the simulation results and related discussions. The paper then concludes in Section 5 with some potential future research directions.

## 2. System Model

**Figure 1:**
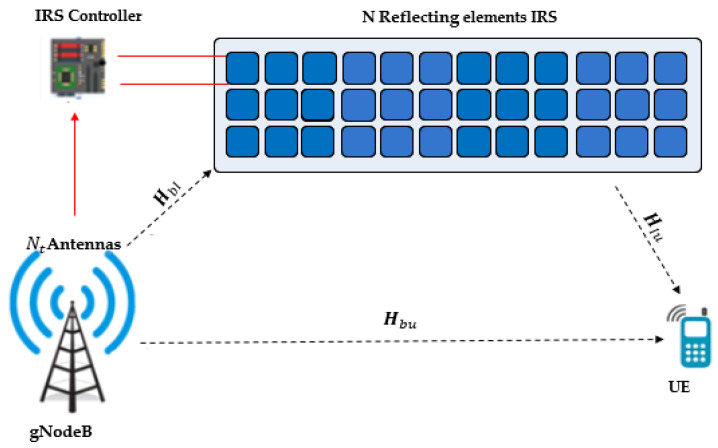
IRS-assisted MISO System Model.

Consider a MISO system where gNodeB of *N_t_* multiple transmitting antennas and the UE with a single antenna are deployed. The communication between the gNodeB and the UE is through intelligently controlled reflecting surfaces. These IRS units are coordinated by a smart software controller, which sets the phase shift for each element. The controller ensures the effective coordination of the reflecting modes of the IRS units. The system model is shown in Figure 1. Table 1 illustrates the parameters that are used throughout the core of this paper.

The received signal is the summation of all reflected Electromagnetic (EM) waves from the smart reflecting surfaces with different phase shifts and the EM waves from the gNodeB to the UE.

**Figure 2:**
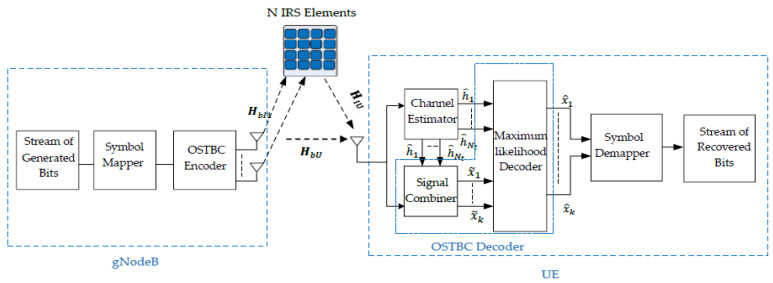
Fully Utilized STBC transceiver with IRS.

Figure 2 shows the detailed fully utilized MISO system that employs the concept of OSTBC and IRS. Fully utilized means that all transmitting antennas and IRS reflecting elements are utilized. As shown in Figure 2, in the STBC encoder, the initial step involves modulating each set of m information bits, where *m* = ${log}_{2}M$ and M is the modulation scheme order. Subsequently, the encoder proceeds with an encoding operation, taking a group of modulated symbols, $x_{1}$, $x_{2}$, …$x_{k}$, and mapping them onto the transmit antennas based on a code matrix. Suppose that an M-ary modulation scheme is deployed. $H_{bI}$ is the channel between the gNodeB and the IRS. $H_{IU}$ is the channel between the UE and IRS. $H_{bU}$ is the channel between the UE and the gNodeB. ${\hat{h}}_{i}$ is the recovered cascaded channel of the transmitting antenna *i*, assuming that the UE has a perfect channel state information. The following section illustrates the OSTBC encoder and decoder in detail.

## 3. Space Time Block Code (STBC)

STBC spreads data symbols across multiple transmit antennas and transmits them over a specific duration. The STBC rate (*R*) is determined by the ratio of the number of modulated symbols manipulated by the encoder (*K*) and the number of transmission time slots (*T_s_*) required to transmit these symbols from multiple antennas [34].

(1) $$R=\frac{K}{T_{s}}$$

Consider a scenario where the signal constellation contains $2^{m}$ points. During each encoding operation, a block of *Km* information bits is mapped onto the signal constellation to select *K* modulated symbols, represented as $\;x_{1}$,$x_{2}$,….$x_{k}$. In this situation, each group of *m* bits corresponds to the selection of a specific signal from the constellation [35]. These k modulated symbols are subsequently encoded using a space-time block encoder, resulting in the generation of parallel signal sequences. The *N_t_* transmitting antennas simultaneously transmit these sequences over *T_s_* discrete time slots. In [36], it is mathematically proven that complex OSTBC with full rate did not exist for more than two transmission antennas. So, the presented work will be inconsistent with this constraint.

### 3.1. Alamouti STBC System Model

Alamouti scheme considers two antennas at the transmitting side and one antenna at the receiving side (2 × 1). In this scheme, a pair of complex information symbols ($x_{1}$ and $x_{2}$) are selected from the symbol mapper (*M*-QAM) constellation to be transmitted from two transmit antennas over two symbol intervals [37]. The code rate in the Alamouti scheme is 1, which is known by full rate diversity. The transmission is carried out in an orthogonal manner, and the following codeword is used in the transmission. The transmission orthogonal symbol matrix X [38] is:

(2) $$X=\left[\begin{array}{cc} x_{1} & {-x}_{2}^{*}\\ x_{2} & x_{1}^{*} \end{array}\right],$$

where in the first transmission period the symbols $x_{1}$ and $x_{2}$ are transmitted simultaneously from the first and second antenna, respectively. In the second transmission period, antenna one transmits the symbol ${-x}_{2}^{*}$ and antenna two transmit the symbol $x_{1}^{*}$. To check the orthogonality, an identity matrix results when multiplying the codeword matrix by its Hermitian ($X^{H}$), where $X^{H}$ is the conjugate transpose of the matrix X [35].

The received baseband signal reflected through the IRS with *N* passive elements can be expressed as [39]:

(3) Y=HX+W,

where $Y\in C^{1\times N_{t}}$ is a row vector for the received signals whose elements are $y_{i}$ which represents the received signal at time slot i. ***H*** is the cascaded fading channel coefficient, which is the summation of the channel gain of the direct link from the transmitting antenna and the reflection from the IRS elements. The cascaded fading channel coefficient from the first transmitting antenna to the receiving antenna is denoted by $h_{1}$, and the cascaded channel between the second transmitting antenna and the receiving antenna is denoted by $h_{2}$. Assuming that the fading coefficients, $h_{1}\;and\;h_{2}$, are constant across two consecutive symbol transmission periods [40], $W\;\in C^{1\times N_{t}}$ is the Additive White Gaussian Noise vector (AWGN) term with zero mean and unit variance.

The received signal Y can be defined as [25]:

(4) $$Y=\sqrt{\frac{E_{s}}{2}}H\left[\begin{array}{cc} x_{1} & -x_{2}^{*}\\ x_{2} & x_{1}^{*} \end{array}\right]+W,$$

***H*** can be defined as [41,42,43]:

(5) $$H=\left[h_{1}\;h_{2}\right]=H_{bU}^{H}+H_{IU}^{H}\varphi H_{bI}$$

(6) $$\varphi =\alpha \;diag(e^{j\theta_{1}},e^{j\theta_{2}},e^{j\theta_{3}},\ldots \ldots e^{j\theta_{N}}),$$

$\varphi \in C^{N\times N}$ is a diagonal matrix of the adjustable phase of the *j^th^* reflector. $\varphi =\alpha \mathrm{diag}(e^{j\theta_{1}},$ $e^{j\theta_{2}},e^{j\theta_{3}},\ldots \ldots e^{j\theta_{N}}$), where *N* is the number of the reflecting elements and *N_t_* is the number of the antennas at the transmitting side.$\theta_{j}$ is a random value from $0^{{}^{\circ}}$ to $90^{{}^{\circ}}$. α is the amplitude coefficient with values from 0 to 1. ***X*** is the transmitted data symbol matrix. $H_{bI}\;\in C^{N\times N_{t}}$ is the channel between the gNodeB and the IRS. $H_{IU}\in C^{N\times 1}$ is the channel between the UE and the IRS. $H_{bU}\in C^{N_{t}\times 1}$ is the channel between the UE and the gNodeB.

$H_{bU}^{H}{,H}_{IU}^{H},\;and\;H_{bI}$ are modeled as Rayleigh flat fading channel, with 𝒞𝒩 (0, 1) distribution, i.e., with zero mean and unit variance. $E_{s}$ is the energy per symbol, which is independent on the number of active transmitting antennas [44,45].

The received signal at the first time slot is:

(7) $$y_{1}=h_{1}x_{1}+h_{2}x_{2},$$

The received signal at the second time slot is:

(8) $$y_{2}=-h_{1}x_{2}^{*}+{x_{1}^{*}h}_{2},$$

In order to predict the transmitted symbol successfully, two different methodologies are deployed at the UE. The first methodology is based on the Maximum Likelihood (ML) estimation decoder only, which depends on the minimum Euclidean distance. Supposing that all the signals in the modulation constellation are equiprobable. The ML decoder chooses a pair of signals ($x_{1},x_{2}$) from the signal constellation to minimize the distance metric over all possible values of ${\hat{x}}_{1}$ and ${\hat{x}}_{2}$ [35]. Hence, the number of steps required to detect the correct symbol by ML is *M*^2^. The process of ML detection can be understood as the act of minimizing the following decision metric (9):

(9) $$d^{2}\left(y_{1},h_{1}{\hat{x}}_{1}+h_{2}{\hat{x}}_{2}\right)+d^{2}\left(y_{2},-h_{1}{\hat{x}}_{2}^{*}+h_{2}{\hat{x}}_{1}^{*}\right)={\left|y_{1}-h_{1}{\hat{x}}_{1}-h_{2}{\hat{x}}_{2}\right|}^{2}+{\left|y_{2}+h_{1}{\hat{x}}_{2}^{*}-h_{2}{\hat{x}}_{1}^{*}\right|}^{2},$$

This decoding scheme necessitates a comprehensive search of all possible pairs and typically, its complexity increases exponentially with the number of transmit antennas and higher modulation orders. On the other hand, the second methodology is based on the combination of the received signals in the first stage. ${\tilde{x}}_{1}\;and\;{\tilde{x}}_{2}$ are extracted by simultaneously solving Equations (7) and (8). Then, the second stage is implemented using the ML detector to apply the decision rules. In this case, ML decoding can be more simplified. The decision statistics of the symbols ${\tilde{x}}_{1}\;and\;{\tilde{x}}_{2}$ can be calculated as follows [35]:

(10) $${\tilde{x}}_{1}=\frac{1}{{\left\|\left.H\right\|\right.}^{2}}(h_{1}^{*}y_{1}+h_{2}y_{2}^{*}),$$

(11) $${\tilde{x}}_{2}=\frac{1}{{\left\|\left.H\right\|\right.}^{2}}(h_{2}^{*}y_{1}-h_{1}y_{2}^{*}),$$

(12) $${\left\|\left.H\right\|\right.}^{2}=\sum_{i=1}^{2}{\left\|\left.h_{i}\right\|\right.}^{2}$$

The likelihood received symbols ${\hat{x}}_{1}$ and ${\hat{x}}_{2}$ will be the nearest estimated symbol in the constellation could be done by minimizing the distance between the decision statistics of the symbol ${\tilde{x}}_{i}$ and the set of all the possible symbols in the constellation $s_{i}\in$ ***S*** [35].

(13) $${\hat{x}}_{i}=arg\underset{s_{i}\in S\;}{\min}{\left|s_{i}-{\tilde{x}}_{i}\right|}^{2}$$

Hence, the number of steps required to detect the correct symbol by ML is 2M. So, the second method will be used through this paper to detect the received symbol, since it requires fewer steps for the ML detector.

### 3.2. OSTBC System Model

In this work also, in a MISO System, four antennas will be at the transmitting side and one antenna will be at the receiving side. The code rate used is $\frac{3}{4}$, meaning that the three symbols will be sent in four different time slots. The transmission is carried out in an orthogonal manner; the following codeword is used in the transmission [40,46,47]. The transmission orthogonal symbol matric X is:

(14) $$X=\left[\begin{array}{cccc} x_{1} & x_{2} & x_{3} & 0\\ -x_{2}^{*} & x_{1}^{*} & 0 & x_{3}\\ x_{3}^{*} & 0 & -x_{1}^{*} & x_{2}\\ 0 & x_{3}^{*} & -x_{2}^{*} & -x_{1} \end{array}\right],$$

To check that this matrix is orthogonal, an identity matrix results from multiplying the matrix X by its Hermitian transpose $X^{H}$. The received signal Y can be defined as:

(15) $$Y=\sqrt{\frac{E_{s}}{2}}H\left[\begin{array}{cccc} x_{1} & x_{2} & x_{3} & 0\\ -x_{2}^{*} & x_{1}^{*} & 0 & x_{3}\\ x_{3}^{*} & 0 & -x_{1}^{*} & x_{2}\\ 0 & x_{3}^{*} & -x_{2}^{*} & -x_{1} \end{array}\right]+W,$$

(16) $$H=\left[h_{1}\;h_{2}\;h_{3}\;h_{4}\right]=H_{bU}^{H}+H_{IU}^{H}\varphi H_{bI},$$

At the receiving side, the four received signals will be combined and then sent to the ML detector. After combining these signals, they are solved simultaneously to get the decision statistics for all the symbols [47].

(17) $${\tilde{x}}_{1}=\frac{1}{{\left\|\left.H\right\|\right.}^{2}}(h_{1}^{*}y_{1}+h_{2}y_{2}^{*}-h_{3}y_{3}^{*}-h_{4}^{*}y_{4})$$

(18) $${\tilde{x}}_{2}=\frac{1}{{\left\|\left.H\right\|\right.}^{2}}(h_{2}^{*}y_{1}-h_{1}y_{2}^{*}+{h_{4}^{*}y}_{3}-h_{3}y_{4}^{*})$$

(19) $${\tilde{x}}_{3}=\frac{1}{{\left\|\left.H\right\|\right.}^{2}}(h_{3}^{*}y_{1}+h_{4}^{*}y_{2}+h_{1}y_{3}^{*}+h_{2}y_{4}^{*})$$

(20) $${\left\|\left.H\right\|\right.}^{2}=\sum_{i=1}^{4}{\left\|\left.h_{i}\right\|\right.}^{2}$$

The likelihood received symbols ${{\hat{x}}_{1},\hat{x}}_{2},$ and ${\hat{x}}_{3}$. ML detector is used to select the closest symbol to the decision statistics of the symbol ${\tilde{x}}_{i}$, as in Equation (13). The system-performance assessment is summarized by deploying the following Algorithm 1.

**Algorithm** **1:** BER calculation for the proposed system
**Inputs:** Number of bits (N_bits), number of bits taken each $T_{s}$ (N_mod), Number of IRS reflecting elements (N), Modulation order (M), QAM modulation, Number of transmitting antennas $N_{t}$$\;(N_{t}$$=2\;“\mathrm{Alamouti}”,\;N_{t}$ = 4 “OSTBC 4 × 1”)1: **for** each E_b_/N_o_ **do**2:  **While** N_bits > N_mod **do**3:    select N_mod = $\log_{2}M$ *x K*4:    Generate modulated symbols $x_{1}$$,x_{2}$$,\ldots .x_{k}$.5:    Compute ***X*** using Equation (2) for Alamouti or Equation (14) for OSTBC6:    Generate $\varphi =\alpha \mathrm{diag}(e^{j\theta_{1}},$$e^{j\theta_{2}},e^{j\theta_{3}},\ldots \ldots e^{j\theta_{N}}$)7:    Compute Y using Equation (4) for Alamouti or Equation (15) for OSTBC8:    Compute $\;{\tilde{x}}_{i}$ using Equations (10) and (11) for Alamouti, or Equations (17)–(19) for OSTBC.9:    **for** each Symbol in ***S* do**10:      ${\hat{x}}_{i}=arg\underset{s_{i}\in S}{\min}{\left|s_{i}-{\tilde{x}}_{i}\right|}^{2}$11:    end **for**12:    Map ${\hat{x}}_{i}$ to the corresponding symbol13:    Demodulate the likelihood ${\hat{x}}_{i}$ into bits14:  end **while**15:  Calculate BER16: end **for****Output**: BER


## 4. Results: Analysis and Discussion

MATLAB R2024 environment was conducted in order to evaluate the system performance for both Alamouti STBC 2 × 1 assisted IRS and as well as OSTBC 4 × 1 assisted IRS. In order to validate the proposed system models, we illustrate a comparative study for altering the number of reflecting elements, as well as the modulation scheme, and the number of the transmitting antennas. The number of antennas in the transmitting side is varied in order to investigate the system’s performance in terms of the diversity gain. On the other hand, the effect of having different sizes of reflecting elements is investigated by deploying two scenarios (16 and 64 IRS reflecting elements). This will help to assess the impact of the number of serving IRS reflecting elements versus the obtained BER. From the perspective of the wireless channel model, the Rayleigh fading channel is used throughout the whole work that is presented in this paper.

Table 2 shows all the simulation parameters that are adjusted throughout all the simulations in this paper.

**Figure 3:**
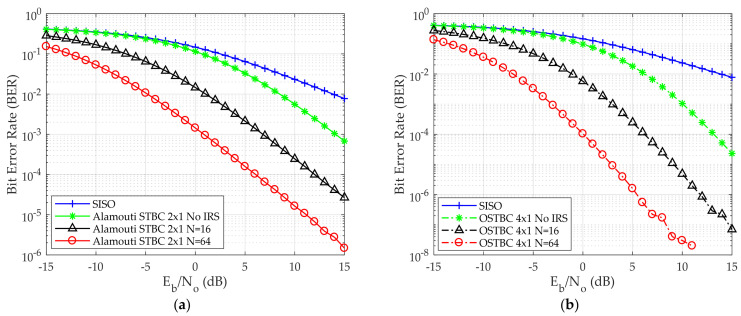
BER versus E_b_/N_o_ for the QPSK modulation scheme (**a**) Alamouti STBC 2 × 1 deployed (**b**) OSTBC 4 × 1 deployed.

Figure 3a,b shows BER versus E_b_/N_o_ for both Alamouti and OSTBC, respectively. Figure 3a validates that the computed QPSK BER of Single Input Single Output (SISO) and Alamouti STBC 2 × 1 is the same as the previously published work in [25].

In Figure 3a, as a point of comparison BER 10^−2^ is taken. E_b_/N_o_ is 14 dB, 8 dB, 1 dB, −5 dB for SISO, Alamouti STBC without IRS, Alamouti STBC with 16 reflecting elements, and Alamouti STBC with 64 reflecting elements, respectively. Furthermore, it has been established that an enhancement in error performance can be attained through the use of IRS and by increasing the number of reflecting elements in the IRS. In Figure 3b four transmitting antennas are used instead of two transmitting antennas, as in the case of Alamouti in Figure 3a. As a result of increasing the number of transmitting antennas, the BER decreases. This is due to the diversity gain between multiple simultaneous antennas where the data has been sent multiple times. When increasing the number of reflecting elements, the BER decreases. At BER 10^−2^, E_b_/N_o_ is 14 dB, 6 dB, −1 dB, −7 dB for SISO, OSTBC without IRS, OSTBC with 16 reflecting elements, and OSTBC with 64 reflecting elements, respectively.

**Figure 4:**
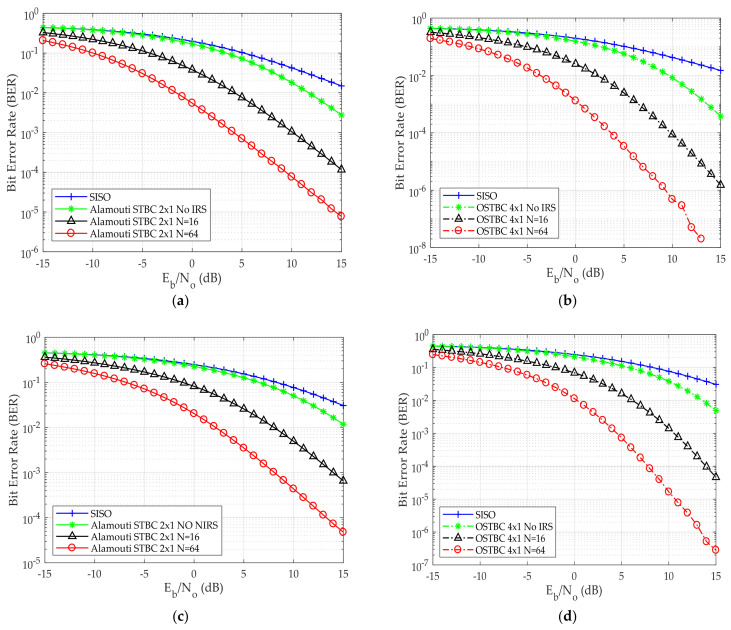

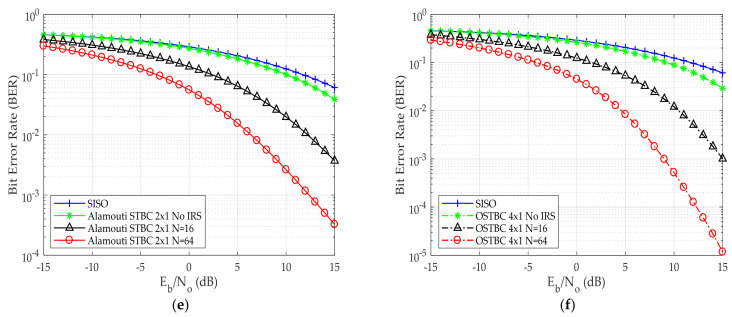
BER versus E_b_/N_o_ (**a**) Alamouti STBC employing 16 QAM scheme (**b**) OSTBC employing 16 QAM scheme (**c**) Alamouti STBC employing 64 QAM scheme (**d**) OSTBC employing 64 QAM scheme (**e**) Alamouti STBC employing 256 QAM scheme (**f**) OSTBC employing 256 QAM scheme.

Different from the previous published work, higher modulation schemes are deployed. Moreover, deploying different IRS reflecting elements is used to reduce the number of transmitting antennas to avoid system complexity at the gNodeB. The BER performance versus E_b_/N_o_ for different numbers of IRS reflecting elements and varying the modulation scheme used for both Alamouti 2 × 1 and OSTBC 4 × 1 is shown in Figure 4.

Figure 4a,b shows the error performance of the proposed system for Alamouti and OSTBC using 16-QAM as the modulation scheme, respectively. To demonstrate the impact of the improvement obtained via the implementation of IRS, the BER is taken to be 10^−2^ for various systems (for the purpose of comparison). It was determined that the required E_b_/N_o_ decreases by approximately 7 dB and 13 dB when using an IRS with 16 and 64 reflecting elements, respectively, in comparison to the proposed system without using IRS. Thus, the deployment of IRS enhances the prediction probability of the received symbol. This is due to the gain of constructive combination of multiple reflective signals from different IRS elements, this is achieved instead of having a more complex system. The complexity analysis will be carried out in our future work.

Figure 4c,d shows BER versus E_b_/N_o_ for Alamouti and OSTBC using 64 QAM. In Figure 4e there is a reduction in the required E_b_/N_o_ of about 2 dB when utilizing two antennas (Alamouti STBC) at the transmitting side instead of using one antenna when taking BER 10^−2^ as a comparative point. In addition, 256 QAM performance for both Alamouti and OSTBC is shown in Figure 4e,f, respectively. To conclude, when employing 16 reflecting elements, the utilization of IRS results in a 7 dB decrease in the required E_b_/N_o_ when compared to the scenario without IRS. Similarly, with 64 reflecting elements, the required E_b_/N_o_ reduced by 13 dB. Remarkably, this improvement remains consistent for all modulation techniques employed, including QPSK, 16 QAM, 64 QAM, and 256 QAM.

Table 3 shows the required E_b_/N_o_ at BER 10^−2^ for the case of Alamouti and OSTBC for all the modulation schemes. It shows that the required E_b_/N_o_ decreases by 7 dB and by 13 dB when deploying the 16 and 64 IRS elements respectively when compared to the non-IRS system for all modulation schemes.

**Figure 5:**
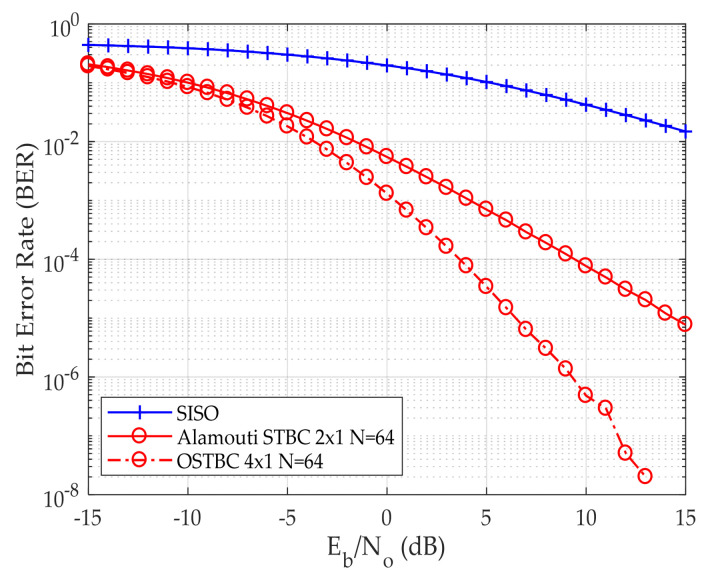
BER for Alamouti STBC and OSTBC (4 × 1) using the 16 QAM scheme.

Figure 5 illustrates the BER versus E_b_/N_o_ for both Alamouti and OSTBC utilizing the 16 QAM technique using 64 reflecting elements for IRS. OSTBC outperforms Alamouti due to the more diversity the system has in order that the received signal will be recognized correctly more than by the Alamouti technique. This is done via the diversity gain between multiple simultaneous antennas where the data has been sent multiple times. In Alamouti the symbol is sent twice, while in OSTBC (4 × 1) the symbol is sent four times. So, the prediction of the symbol will be improved. At BER 10^−4^, E_b_/N_o_ is 4 dB, and 10 dB when utilizing two antennas (Alamouti 2 × 1), and four antennas (OSTBC 4 × 1) at the transmitting side, respectively. Thus, the required E_b_/N_o_ reduced by 6 dB. The increase of the number of transmitting antennas directly improves symbol detection, resulting in a decrease in BER. This is due to diversity gain.

**Figure 6:**
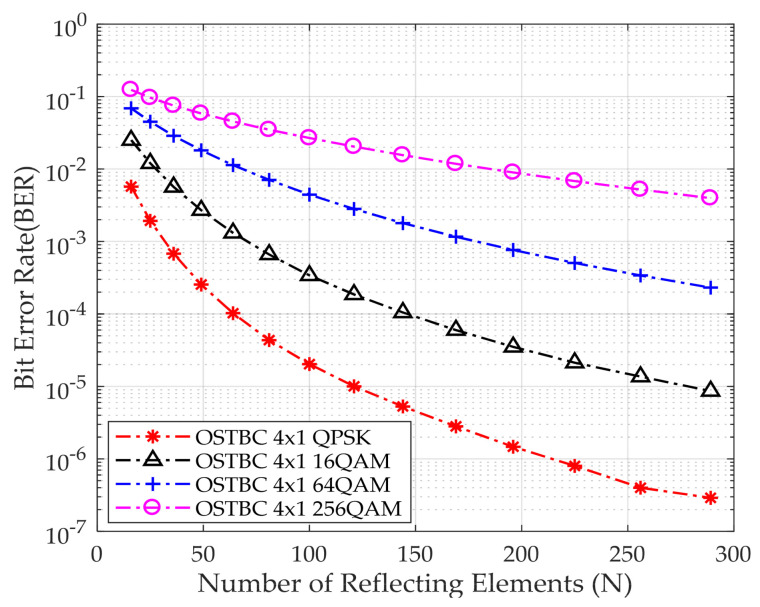
BER performance versus the number of reflecting elements at E_b_/N_o_ equal to 0 dB.

Moreover, the system has been simulated for constant E_b_/N_o_ equal to 0 dB while investigating the effect of having different numbers of reflecting elements of the IRS, to evaluate the effect of increasing the number of reflecting surfaces on BER. It is assumed that the IRS is in a square orientation. The number of reflecting elements on one side ranges from 4 to 17, incrementing by 1. Figure 6 demonstrates that increasing the number of reflecting elements leads to an enhancement in detecting the received bits, which leads to less BER in the system. Moreover, when employing a higher modulation order, the BER increases as a result of the increased challenge in detecting the received bits.

Table 4 shows BER for different reflecting elements and modulation schemes at E_b_/N_o_ equal to zero. In the case of QPSK, by increasing the number of reflecting elements from 49 to 100, the BER decreases by approximately a decade, and by increasing the number of reflecting elements more than 100, the BER decreases by around a decade. Moreover, for 100 reflecting elements at fixed E_b_/N_o_ equal to zero, the BER increases by almost a decade for different modulation schemes. This is due to the challenge of detecting the received bits.

**Figure 7:**
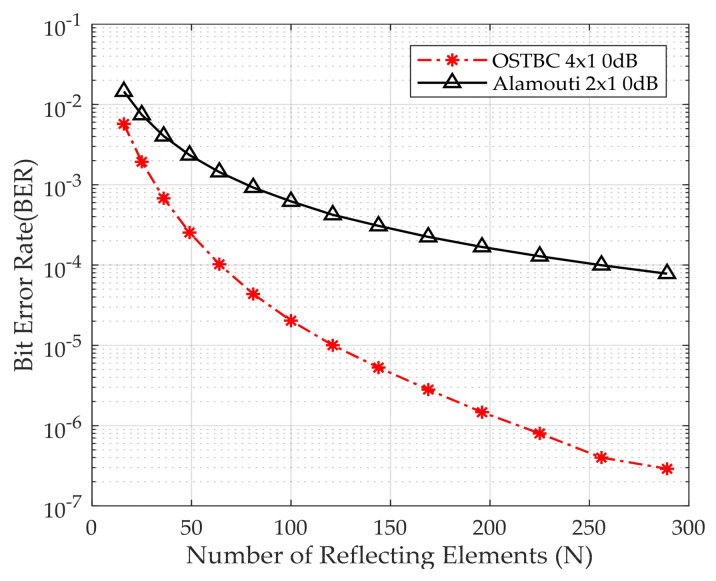
BER performance versus the number of reflecting elements.

Figure 7 shows the BER versus the number of reflecting elements for Alamouti and OSTBC at E_b_/N_o_ 0 dB using QPSK. BER decreases by the increase of the reflecting elements of the IRS. The BER is 4 × 10^−7^, 10^−4^ for OSTBC, and Alamouti, respectively, when using 256 reflecting elements. OSTBC, the system with greater diversity, outperforms Alamouti, as it increases the precision of recognizing the received signal compared to the Alamouti technique. Thus, the number of antennas used at the transmitting side is directly proportional with the accuracy of the system.

**Figure 8:**
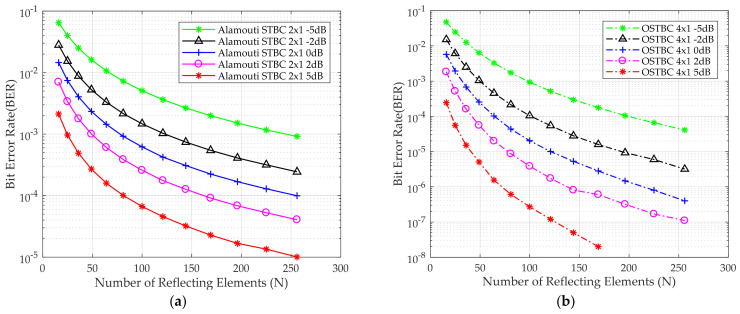
BER for the QPSK modulation scheme versus the number of IRS reflecting elements (**a**) Alamouti STBC 2 × 1 deployed (**b**) OSTBC 4 × 1 deployed at different E_b_/N_o_.

Figure 8a,b shows the BER performance for Alamouti and OSTBC versus the number of reflecting elements, respectively, for different E_b_/N_o_ using QPSK as a modulation scheme as a proof of concept that the BER decreases by increasing of E_b_/N_o_. Moreover, by boosting the energy of the received signal via an increase in the number of reflecting elements of the IRS, BER decreases.

**Figure 9:**
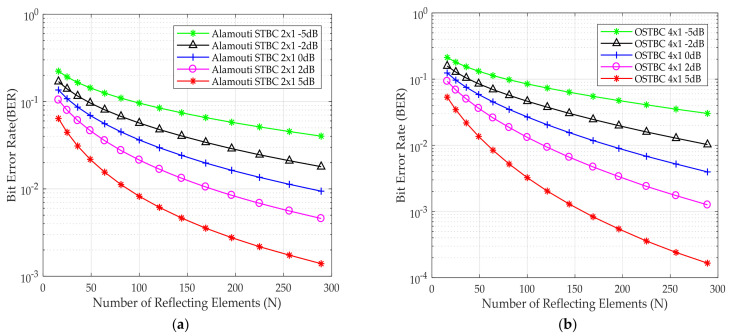
BER for the 256 QAM modulation scheme versus the number of IRS reflecting elements (**a**) Alamouti STBC 2 × 1 deployed (**b**) OSTBC 4 × 1 deployed at different E_b_/N_o_.

Figure 9a,b shows the BER performance for Alamouti and OSTBC as a function of the number of reflecting elements, respectively, for different values of E_b_/N_o_ using 256 QAM as a modulation scheme. BER decreases by increasing the reflecting elements.

## 5. Conclusions

In this paper, an IRS-assisted MISO system using the OSTBC scheme is studied. The performance of the proposed system was assessed by conducting simulations with different operation scenarios, including the number of transmitting antennas, the number of IRS reflecting elements, and the higher modulation schemes. The simulation outcomes provide evidence that the combination of OSTBC and IRS leads to notable improvements in terms of BER. The utilization of IRS with 16 and 64 reflecting elements yields a significant 7 dB and 13 dB reduction in the required E_b_/N_o_ when compared to the non-IRS scenario. This improvement remains consistent across various modulation schemes, including QPSK, 16 QAM, 64 QAM, and 256 QAM. Moreover, the utilization of four transmitting antennas instead of two reduces the required E_b_/N_o_ by 6 dB. This reduction can be attributed to the diversity gain achieved through the increased number of transmitting antennas. Moreover, when deploying IRS, it outperforms the system without IRS. This is due to the constructive interference resulted by the reflections from the IRS. So, the overall system BER will decrease or the required E_b_/N_o_ will decrease, which will let the system go more and more green in terms of technology. In addition, by increasing the number of reflecting elements, the number of transmitting antennas will decrease to avoid system complexity at the gNodeB. Moreover, the IRS is low cost in comparison with antennas, so the overall cost of the system will decrease. In the future work, the implementation of the system will be considered to compare the experimental results with the simulated results. The optimization of the IRS phase shifts will also be considered in the future work. Moreover, in the future work, OSTBC use in IRS-aided massive MIMO systems will be studied.

## Figures and Tables

**Table 1:** List of parameters with description.

Parameter	Description
α	The amplitude coefficient with values from 0 to 1
$\theta_{j}$	$\mathrm{IRS}\;\mathrm{phase}\;\mathrm{angle}\;(\mathrm{value}\;\mathrm{from}\;0^{{}^{\circ}}$$\mathrm{to}\;90^{{}^{\circ}}$)
φ	Diagonal matrix of the adjustable phase
$E_{s}$	Energy per symbol
***H***	The cascaded fading channel coefficient
$H_{bI}$	The channel between the gNodeB and the IRS (*N* × *N_t_*)
$H_{bU}$	The channel between the UE and the gNodeB (*N_t_* × 1)
$H_{IU}$	The channel between the UE and the IRS (*N* × 1)
$h_{i}$	The cascaded channel between transmitting antenna *i* and IRS
${\hat{h}}_{i}$	The recovered cascaded channel of the transmitting antenna *i*
*K*	The number of modulated symbols during each *T**_s_***
*M*	Modulation order
*m*	Number of information bits
*N*	Number of IRS reflecting elements
*N_t_*	Number of transmitting antennas
W	Additive White Gaussian Noise Vector (AWGN) (1 × *N_t_*)
*R*	Space time block code rate
***S***	Set of all possible symbols in the constellation
$s_{i}$	Symbol i in the set of all possible symbols in the constellation ***S***
*T_s_*	The number of transmission time slots
X	The transmission orthogonal symbol matrix
$X^{H}$	The conjugate transpose of the matrix ***X*** (***X*** Hermitian)
*x*	Modulated Symbol
$\hat{x}$	The likelihood received symbol
$\tilde{x}$	Decision statistics of the symbol
Y	The row vector for the received signals (1 × *N_t_*)
$y_{i}$	The received signal at time slot *i*

**Table 2:** Simulation parameters.

Simulation Parameters	Value(s)
Modulation scheme	QPSK, 16-QAM, 64-QAM, 256-QAM
Number of transmitting antennas (*N_t_*)	2 (Alamouti), 4 (OSTBC)
Number of receiving antennas	1
Number of simulated bits	10^8^
Channel models	Rayleigh flat fading, with 𝒞𝒩 (0, 1)
Number of IRS elements (N)	16, 64

**Table 3:** E_b_/N_o_ for Alamouti and OSTBC at BER = 10^−2^ for different modulation schemes.

M_QAM	Alamouti			OSTBC		
	No IRS	N = 16	N = 64	No IRS	N = 16	N = 64
**QPSK**	8 dB	1 dB	−5 dB	6 dB	−1 dB	−7 dB
**16 QAM**	11 dB	4 dB	−2 dB	9 dB	2 dB	−4 dB
**64 QAM**	15 dB	8 dB	2 dB	13 dB	6 dB	0 dB
**256 QAM**	N/A	12 dB	6 dB	N/A	10 dB	4 dB

**Table 4:** OSTBC BER for different reflecting elements and modulation schemes at E_b_/N_o_ = 0 dB.

	N	49	100	144	256	289
M_QAM						
**QPSK**		2.50 × 10^−4^	2.03 × 10^−5^	5.29 × 10^−6^	4.00 × 10^−7^	2.90 × 10^−7^
**16 QAM**		2.70 × 10^−3^	3.40 × 10^−4^	1.05 × 10^−4^	1.37 × 10^−5^	8.64 × 10^−6^
**64 QAM**		1.80 × 10^−2^	4.40 × 10^−3^	1.79 × 10^−3^	3.40 × 10^−4^	2.31 × 10^−4^
**256 QAM**		5.80 × 10^−2^	2.68 × 10^−2^	1.56 × 10^−2^	5.20 × 10^−3^	4.00 × 10^−3^

## Data Availability

Data are contained within the article.
